# Impact of the microcystic, elongated, and fragmented invasion pattern on prognosis in endometrial carcinoma: a comprehensive meta-analysis

**DOI:** 10.3389/fonc.2025.1527324

**Published:** 2025-06-25

**Authors:** Li Zhou, Chengyao Li, Jianguo Zhao, Pu Li, Pengpeng Qu

**Affiliations:** ^1^ Clinical School of Obstetrics and Gynecology Center, Tianjin Medical University, Tianjin, China; ^2^ Department of General Gynecology, Tianjin Central Hospital of Obstetrics and Gynecology, Tianjin, China; ^3^ Department of Gynecological Oncology, Tianjin Central Hospital of Obstetrics and Gynecology, Tianjin, China

**Keywords:** microcystic, elongated, and fragmented (MELF) pattern, endometrial carcinoma, lymph node metastasis, molecular classification, prognosis

## Abstract

The prognostic significance of the microcystic, elongated, and fragmented (MELF) invasion pattern in endometrial carcinoma (EC) has not been fully elucidated. This study conducted a systematic search across five electronic databases (PubMed, EMBASE, Cochrane, Web of Science, and the Chinese National Knowledge Infrastructure (CNKI)) from inception to April 2025. Assessment focused on overall survival (OS), disease-free survival (DFS), lymph node metastasis (LNM), and recurrence rate (RR) as primary outcomes. Meta-analysis calculations of overall odds ratios (ORs) and hazard ratios (HRs) with corresponding 95% confidence intervals (CIs) elucidated the impact of the MELF pattern on clinical outcomes. Analysis of 18 studies involving 5587 participants revealed a significant correlation between the presence of MELF pattern and heightened LNM incidence (OR 3.52, 95% CI: 2.17–5.71, p < 0.001). Univariate analysis indicated a notable inverse relationship between MELF pattern and OS (HR 2.31, 95% CI: 1.67–3.21, p < 0.001), as well as DFS (HR 1.67, 95% CI: 1.20–2.30, p = 0.002). In multivariate analysis, however, this association did not achieve statistical significance (for OS, HR 1.54, 95% CI: 0.99–2.41, p = 0.056; for DFS, HR 1.25, 95% CI: 0.90–1.74, p = 0.191). The findings of this meta-analysis demonstrated that the MELF pattern was linked to elevated risk of LNM and poorer OS and DFS outcomes. The correlation was influenced by various factors including surgical interventions and adjuvant therapies. While potentially increasing the risk of recurrence, the findings did not demonstrate statistical significance (OR 1.15, 95% CI: 0.61–2.16; p = 0.669).

## 1 Introduction

Endometrial carcinoma (EC) has become one of the most common gynecologic malignancies in recent years, attributed to an aging population and escalating obesity rates (1). Endometrioid endometrial carcinoma (EEC) constitutes approximately 80% of all EC cases, typically presenting as low-grade, early-stage tumors with favorable clinical outcomes. Despite this generally positive outlook, approximately 8% to 10% of cases experience recurrences and distant metastases, even at initial stages (2, 3).

The microcystic, elongated, and fragmented (MELF) pattern, initially described by Murray in 2003, represents a distinct histological profile characterized by microcystic, elongated or clustered glands lined by flattened epithelial cells penetrating the myometrium (4). This pattern manifests as an infiltrative morphology at the tumor invasion front, particularly in the deep muscular layer, indicating increased invasive potential. Over the past decade, the MELF pattern has generated significant discussion regarding its molecular and clinicopathologic features (5, 6). Studies (7, 8) have associated MELF pattern with aggressive phenotypes, including deep myometrial infiltration (DMI), large tumor size, lymphovascular space involvement (LVSI), and lymph node metastasis (LNM). However, the precise prognostic implications for patient survival and disease recurrence require further clarification.

In 2013, The Cancer Genome Atlas (TCGA) research network utilized comprehensive molecular data to categorize EC into four distinct prognostic subgroups: polymerase-e ultramutated (POLEmuted), microsatellite instability hypermutated (MSI-H), copy-number low (CNL), and copy-number high (CNH) (9). These molecular classifiers provide insights into tumor behavior and treatment response beyond traditional clinicopathological factors. The FIGO 2023 EC staging system has incorporated this molecular classification approach. The integration of clinicopathological and molecular parameters enhances risk assessment methods, enabling more precise treatment decisions. Several studies have identified distinct molecular classifications within EEC exhibiting the MELF invasion pattern (10–12). The distribution of the four molecular subgroups within tumors showing the MELF invasion pattern and their prognostic implications, however, remain uncertain.

This research aims to synthesize current literature regarding the prognostic significance of the MELF pattern and its association with recurrence in EEC patients. To address heterogeneity inherent in reviewed studies, stringent inclusion criteria have been applied to ensure reliable findings and to elucidate the predictive potential for clinical outcomes. Additionally, a comprehensive literature review was conducted to investigate associations between the MELF invasion pattern and molecular classification in EEC.

## 2 Methods

### 2.1 Search strategy

This systematic review and meta-analysis follows the Preferred Reporting Items for Systematic Reviews and Meta-Analyses (PRISMA) guidelines. The study is registered in the International Prospective Register of Systematic Reviews (CRD42023430435). Ethical approval and patient consent were not required as this research synthesizes data from published literature. A systematic literature search was performed using PubMed, EMBASE, Cochrane, Web of Science, and the Chinese National Knowledge Infrastructure (CNKI) databases, published from inception to April 2025, restricting publications to those available in English and Chinese. The search terms combined both text and MeSH entries: “Endometrial Neoplasm” OR “Neoplasm, Endometrial” OR “Endometrial Carcinoma” OR “Endometrial Cancer” AND “Microcystic, Elongated, and Fragmented” OR “MELF” OR “MELF invasion”. The comprehensive search strategy for PubMed can be found in the **Supplementary Material 1**. Two authors (ZL and LCY) independently conducted the research, resolving any discrepancies through discussion with a third author (ZJG). We reviewed all potentially relevant studies regardless of primary outcome. Additionally, we manually examined the bibliographies of significant studies for additional relevant articles.

### 2.2 Inclusion and exclusion criteria

Selected studies examined the prognostic impact of MELF invasion in EC patients, distinguishing between MELF-positive (MELF+) and MELF-negative (MELF-) cases. The assessed outcomes included lymph node metastasis(LNM), overall survival (OS), disease-free survival (DFS), and recurrence rate (RR). All included studies were retrospective in design. Studies were excluded based on the following criteria: unavailability of full text or extractable data; non-original research (case reports, abstracts, conference papers, reviews, or meta-analysis); redundant data; animal studies; sample sizes fewer than 50 or limited to a single stage or grade; and studies lacking accessible survival data despite presenting survival curves.

### 2.3 Data extraction and quality evaluation

Initial screening of titles and abstracts was performed by LCY and ZL, followed by full-text review of studies meeting inclusion criteria. Discrepancies were resolved through consultation with a third evaluator (ZJG). Extracted data included country of origin, publication year, participant numbers, patient sources, follow-up duration, hazard ratios (HR) and 95% confidence intervals (CI) for OS and DFS, odds ratios (OR) and 95% CI for LNM and relative risk (RR). Clinicopathological factors were also recorded, including tumor grades, FIGO stage, deep myometrial infiltration(DMI), cervical stromal invasion (CSI), and lymphovascular space invasion (LVSI). Primary outcomes were LNM, OS, DFS, and RR. Two reviewers assessed the risk of publication bias according to PRISMA guidelines.

### 2.4 Data synthesis and statistical analysis

The meta-analysis calculated ORs and HRs with corresponding 95% CIs to evaluate the impact of the MELF pattern on clinical outcomes. Study heterogeneity was assessed using the Cochran Q test and Higgins’ *I^2^* statistic. Fixed-effects models were implemented when *P* > 0.05 and *I^2^* < 50%, assuming random variability in results. Random-effects models were employed when *P* < 0.05 and *I^2^* > 50%, accounting for genuine differences between studies and providing more conservative effect estimates. Publication bias was evaluated through visual inspection of funnel plots and Egger’s tests. Meta-regression, sensitivity analysis, and subgroup analysis were conducted to identify sources of heterogeneity. All statistical analyses were performed using STATA (version 15.0).

## 3 Results

### 3.1 Search results and characteristics of eligible studies

**Figure 1:**
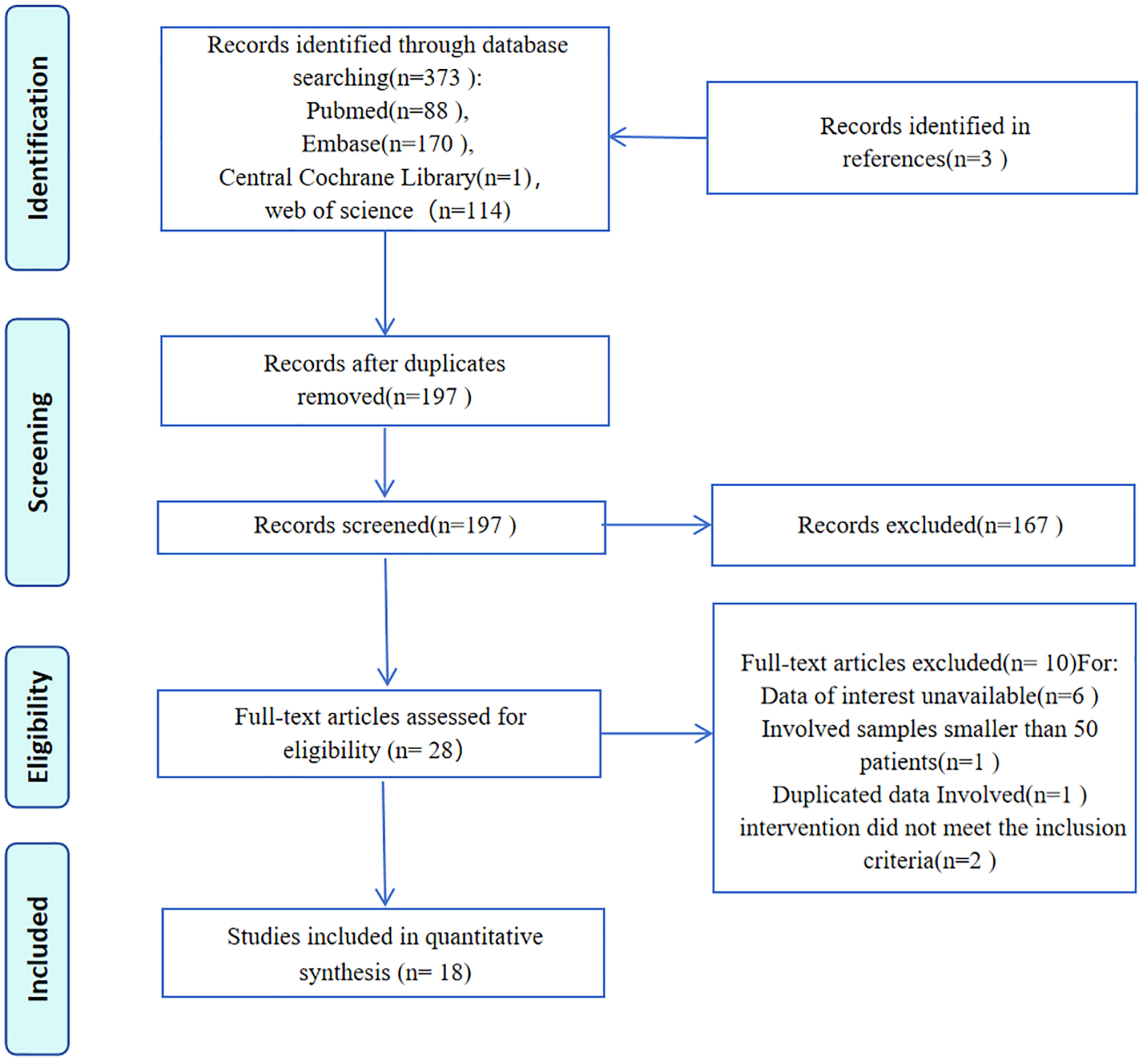
Flow diagram of literature searching and study selection.

The systematic literature search identified 373 entries, from which 18 retrospective studies involving 5,587 participants were selected (**Figure 1**). These studies, conducted between 2013 and 2022, reported case numbers ranging from 51 to 512, with follow-up duration varying from 18 to 135 months. The studies originated from seven countries: Turkey (6 studies) (8, 13–17), China (4 studies) (18–21), the USA (2 studies) (6, 22), Spain (2 studies) (23, 24), Japan (2 studies) (5, 25), the Netherlands (1 study) (26), and Sweden (1 study) (27). Evaluation of MELF infiltration in EC utilized hematoxylin and eosin (HE) stained sections to identify positive cases. Quality assessment using the Newcastle-Ottawa Scale (NOS) revealed scores above 6 for all included studies, indicating high methodological quality (**Table 1**).

**Table 1:** Baseline characteristics of included studies.

Author	Year	Country	Patient source	Sample size	MELF+	outcome	Study design	NOS score
Euscher (6)	2013	USA	American	304	146	LNM	Retrospective	7
Dogan Altunpulluk (13)	2014	Turkey	European	121	38	LVSI, LNM, RR	Retrospective	8
Roma (22)	2015	USA	American	589	59	OS, DFS	Retrospective	6
Kihara (5)	2017	Japan	Asian	479	45	LVSI, DFS	Retrospective	7
Espinosa (23)	2017	Spain	European	101	37	LVSI	Retrospective	8
Sanci (8)	2017	Turkey	European	55	19	OS, DFS, RR	Retrospective	6
Li YM (18)	2018	China	Asian	51	14	LVSI, LNM	Retrospective	7
Eriksson (27)	2018	Sweden	European	850	197	LNM	Retrospective	7
Han HY (20)	2019	China	Asian	179	28	LVSI, LNM, OS, DFS	Retrospective	7
Oge, T (14).	2019	Turkey	European	102	28	LVSI	Retrospective	8
Ruz-Caracuel (24)	2019	Spain	European	258	18	RR	Retrospective	6
OZGUL (15)	2020	Turkey	European	276	24	LNM, RR, DFS	Retrospective	7
Hu CF (21)	2021	China	Asian	512	66	RR, DFS, LNM, RR	Retrospective	7
Yamamoto (25)	2021	Japan	Asian	208	23	OS, DFS, RR	Retrospective	8
Altındağ (16)	2022	Turkey	European	233	51	LVSI, OS, DFS, RR	Retrospective	7
Heerik (26)	2022	Netherlands	European	979	128	LVSI, RR	Retrospective	8
Okcu (17)	2022	Turkey	European	101	29	LVSI, OS, DFS, RR	Retrospective	7
Song JH (18)	2022	China	Asian	189	34	LVSI, LNM, RR	Retrospective	7

LVSI, lymph-vascular space invasion; LNM, lymph node metastasis;RR,recurrence rate; OS, overall survival; DFS, disease-free survival.

### 3.2 Rate of MELF infiltration pattern

The pooled incidence of MELF invasion across all 18 studies (5,587 patients) was 20.4% (95% CI: 16.3%-24.6%).

### 3.3 Molecular classification of EC with MELF pattern

Three studies (10–12) examined the molecular classification of 224 EC patients with MELF invasion. Four molecular subtypes were identified within ECs exhibiting the MELF pattern with the following distribution: 62.1% (95% CI: 55.7%-68.4%) CNL, 23.4% (95% CI: 17.9%-28.9%) MSI-H, 9.4% (95% CI: 3.9%-14.8%) CNH, and 3.4% (95% CI: 0%-6.8%) POLE-mutated.

### 3.4 The association between MELF pattern and clinicopathological features

**Table 2** details the correlation between MELF pattern and various clinicopathological characteristics of EEC. Significant associations were identified between MELF pattern presence and several factors: DMI, LNM, LVSI, CSI, and advanced FIGO stages. DMI was observed in 57.7% of MELF-positive patients compared to 22.3% of MELF- patients (OR: 4.82, 95% CI: 3.10-7.50, *P* < 0.001). CSI was present in 19.4% of MELF+ patients compared to 9.1% of MELF- patients (OR: 2.56, 95% CI: 1.97-3.33, *P* < 0.001). Advanced disease was found in 57.7% of MELF-positive patients compared to 22.3% of MELF-negative patients (OR:3.845,95%CI:2.088-7.077, P < 0.001). Funnel plot analysis and Egger’s tests confirmed minimal publication bias.

**Table 2:** Associations between the MELF pattern and clinicopathological features in patients with EC.

clinicopathological features	Studies(n)	Patients(n)	MELF+			MELF-			OR( 95%CI )	P value	Heterogeneity (I2)	Egger’s test (P value)
			Mevent	Mnoevent	Mtotal	Nevent	Nnoevent	Ntotal				
Myometrial Invasion (≥ 1/2 vs. <1/2)	13	3242	367	269	636	582	2024	2606	4.82(3.098-7.500)	0.000	76.10%	0.360
Cervical Stroma Involvement	10	2995	106	439	545	224	2226	2450	2.562(1.972-3.330)	0.000	35.90%	0.113
Histological Grade(G1-2 Vs G3)	12	3488	601	76	677	2338	473	2811	2.083(1.097-3.957)	0.025	67.40%	0.128
FIGO Stage (III-IV vs. I-II)	9	2302	114	389	503	161	1638	1799	3.845(2.088-7.077)	0.000	77.20%	0.146

FIGO, Federation of International of Gynecologists and Obstetricians; OR, Odds Ratio.

**Figure 2:**
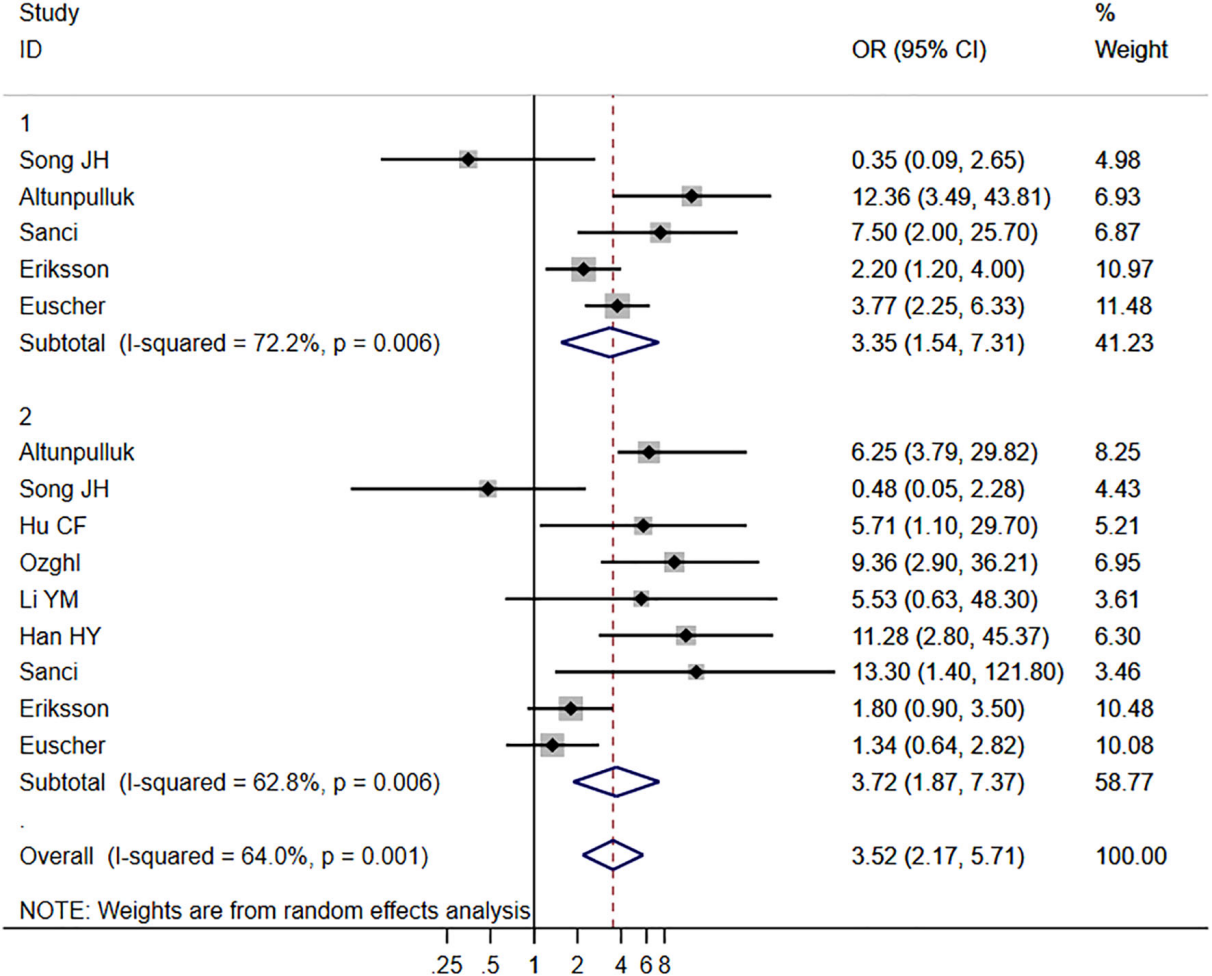
Forest plots of the potential relationships of MELF invasion pattern with lymph node metastasis (LNM).

Ten studies (4, 13, 15, 16, 18–21, 24, 26) provided data on the relationship between MELF and LVSI, while nine studies (7, 13, 15, 18–22, 27) focused on LNM. Using a random-effects model (*I^2^* = 67.6% and 62.8%, respectively), significant associations were found between MELF pattern presence and both LVSI (OR 6.44, 95% CI: 4.82-8.59, *P* < 0.001) and LNM (OR 3.52, 95% CI: 2.17-5.71, *P* < 0.001). These findings are illustrated in **Figure 2**. Sensitivity analysis confirmed the stability of these results. Meta-regression identified patient ethnicity (Asian or European) as a significant source of heterogeneity (*P* < 0.01). Subgroup analyses consistently demonstrated a strong association between MELF and LNM across multiple parameters: in both univariate and multivariate analyses (OR 3.52, 95% CI: 2.17-5.71, *P* < 0.01), across patient ethnicities (OR 3.72, 95% CI: 1.87-7.37, *P* < 0.01), and in studies with sample sizes exceeding 150 (OR 3.72, 95% CI: 1.87-7.37, *P* < 0.001).

### 3.5 The association between MELF and survival

Six studies (8, 16, 17, 20, 22, 25) examining the relationship between MELF invasion pattern and OS exhibited minimal heterogeneity (*I^2^* = 0.0%, *P* = 0.78), permitting application of a fixed-effects model. Meta-analysis using univariate analysis revealed a significant negative correlation between MELF pattern and OS (HR 2.31, 95% CI: 1.67–3.21, *P* < 0.001). In multivariate analysis, this correlation approached but did not achieve statistical significance (HR 1.54, 95% CI: 0.99–2.41, *P* = 0.056), suggesting modulation by factors such as surgical approach and adjuvant therapy.

**Figure 3:**
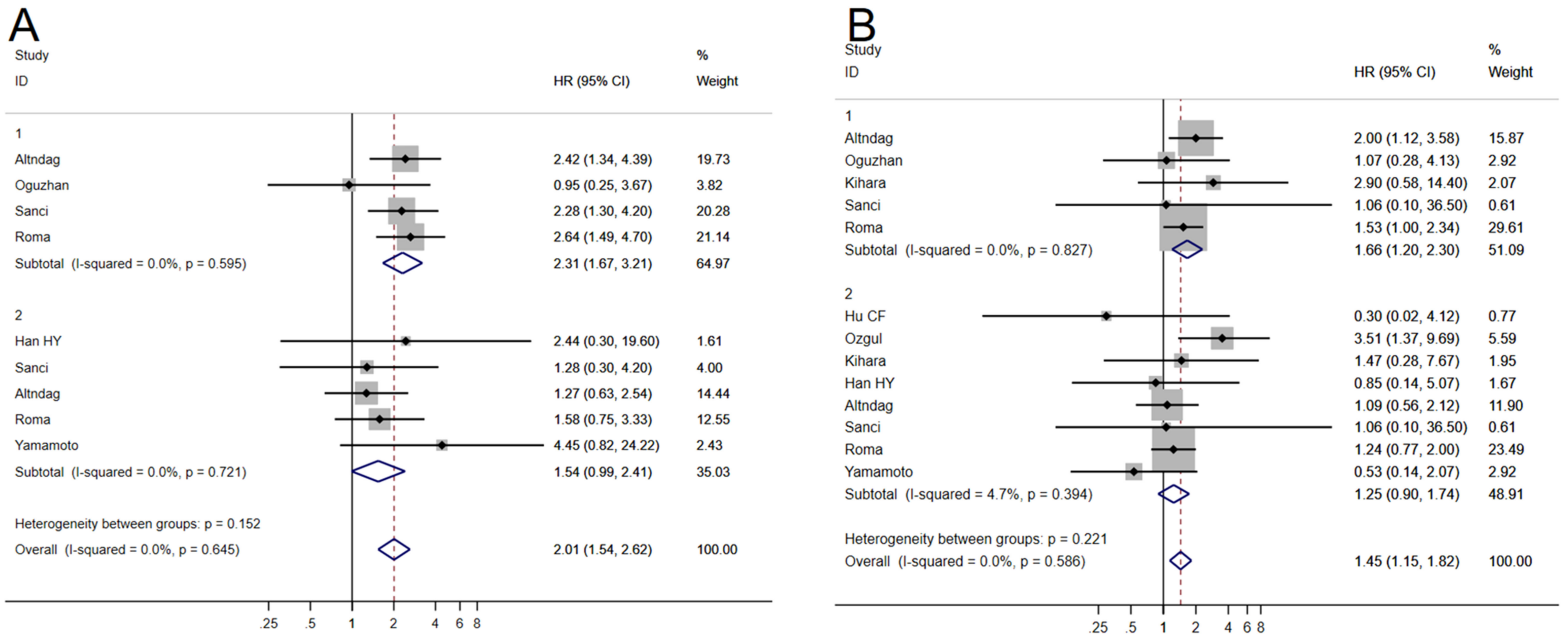
Forest plots of the potential relationships of MELF invasion pattern with overall survival (OS) **(A)**, and disease-free survival (DFS) **(B)**.

Nine studies (5, 8, 12–14, 17–19, 22) evaluating DFS demonstrated minimal heterogeneity (*I^2^* = 0.0%, *P* = 0.495), warranting use of a fixed-effects model. Similar to OS findings, MELF invasion was associated with poorer DFS in univariate analyses (HR 1.67, 95% CI: 1.20–2.30, *P* < 0.01) but not in multivariate analysis (HR 1.25, 95% CI: 0.90–1.74, *P* = 0.20) (**Figure 3**). Sensitivity analyses confirmed result stability across different analytical scenarios that addressed potential biases within existing studies.

### 3.6 The association between MELF and recurrence

**Figure 4:**
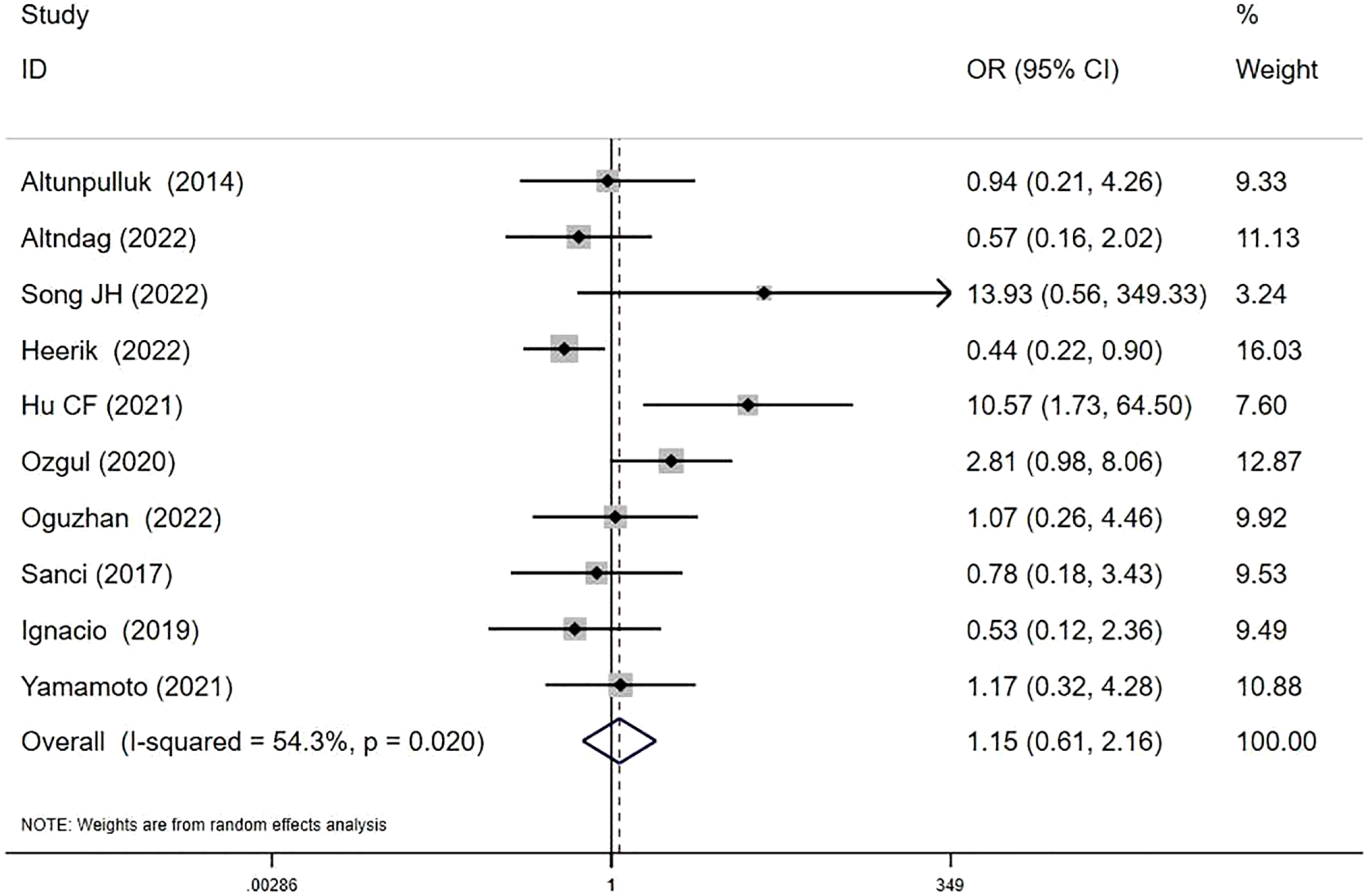
Forest plots of the potential relationships of MELF invasion pattern with recurrence.

Ten studies investigated MELF and recurrence relationships (8, 13, 15–18, 21, 24–26). RRs were 7.91% (37/468) in MELF+ patients versus 8.30% (199/2398) in MELF- patients. The pooled analysis suggested a potential, though statistically non-significant, increase in recurrence risk with MELF infiltration (OR 1.15, 95% CI: 0.61-2.16; *I^2^* = 54.3%, *P* = 0.67) (**Figure 4**). Meta-regression analysis identified patient ethnicity (Asian or European descent) as a significant source of heterogeneity (*P* < 0.05).

### 3.7 Publication bias

Publication bias assessment employed funnel plots comparing each study’s effect size with its standard error, with asymmetry evaluated using Egger’s tests. All funnel plots demonstrated symmetry. Egger’s tests revealed no significant publication bias for studies analyzing OS (*P* = 0.59), DFS (*P* = 0.44), LNM (*P* = 0.16), or RRs (*P* = 0.06). These findings are presented in **Supplementary Material 2**.

## 4 Discussion

MELF pattern represents a distinct histological manifestation of myometrial invasion predominantly observed in low-grade EEC. This meta-analysis demonstrated significant associations between MELF pattern and established adverse prognostic factors in EC, including DMI, LVSI, LNM, CSI, and advanced FIGO stages. These findings suggest that MELF pattern serves as an indicator of invasive tumor phenotype and potentially influences clinical outcomes in patients with EEC. Mechanistic studies have demonstrated elevated expression of invasive markers such as cyclin D1, P16, fascin, and MMP2 in MELF areas, indicating association with epithelial-mesenchymal transformation (EMT) (28). Our meta-analysis reveals that EECs with MELF pattern exhibit significantly higher rates of LNM and LVSI compared to those without MELF. These findings underscore the importance of detecting MELF pattern, particularly in patients with low-grade EEC, as this information directly impacts surgical staging decisions. Hertel and colleagues noted that in cases with MELF, LNM may present subtly as isolated tumor cells (ITCs) or small aggregations resembling histiocytes, which could be inadvertently overlooked (29). Considering these clinicopathological associations, thorough assessment for occult LNM is essential in the surgical staging of low-grade EEC. The relationship between MELF and different categories of lymph node involvement (ITCs, micrometastasis, or macrometastasis) remains incompletely characterized due to limited available studies. Further well-stratified research is necessary to elucidate the correlation between MELF infiltration and various patterns of lymph node involvement.

Survival analysis findings across studies have shown inconsistencies. Guo’s meta-analysis (28) reported no significant correlation between MELF invasion and RR, OS, or DFS, contradicting our findings. This discrepancy results from methodological differences in inclusion criteria. Our meta-analysis excluded studies with fewer than 50 participants, those limited to a single stage or grade, and those presenting only survival curves without accessible underlying data, thereby enhancing statistical validity. In our analysis, univariate assessments revealed a significant negative correlation between MELF pattern and both OS and DFS. However, multivariate analysis failed to demonstrate statistical significance, suggesting that this correlation is modulated by confounding factors such as surgical approach or adjuvant therapy. Alternatively, MELF invasion may interact with multiple adverse prognostic factors rather than functioning as an independent prognostic indicator. Given the limited number of available studies, large-scale prospective investigations are needed to validate these observations. Our meta-analysis found no significant association between MELF pattern and RR (OR 1.15, 95% CI: 0.61-2.16; *I^2^* = 54.3%, *P* = 0.67). Heterogeneity in MELF pattern incidence across studies may account for these results.

Research on molecular characterization of EC exhibiting MELF pattern remains limited. The distribution of molecular subgroups across the three available studies shows relative consistency, with CNL representing the predominant subgroup, followed by MSI-H and CNH. Zhang et al. identified a significantly higher prevalence of mismatch repair-deficient (MMRd) proteins in the MELF group compared to the non-MELF group, suggesting a potential association with tumor immunological responses. However, other studies have not corroborated this finding. In the MELF-infiltrating MMRd subtype, three studies examined the absence patterns of the four MMR proteins. Loss of MLH1–PMS2 coexpression was identified as the most frequent MMRd pattern, followed by loss of MSH6–MSH2 coexpression.

Patients with MELF infiltration demonstrate differential recurrence risks across molecular subgroups. Two studies (10, 11) documented varying RRs, with the CNH subgroup showing the highest incidence (5.5%, 55.6%), followed by CNL (2.5%, 27.7%) and MSI-H (1.5%, 12.5%) subgroups. Notably, no recurrences were observed in the POLEmut subgroup. Regarding progression-free survival, one study (10) found that the CNH subgroup had the poorest outcomes, followed by CNL, while POLEmut and MSI-H subgroups demonstrated more favorable prognosis. The literature on prognostic implications of molecular subgroups in MELF infiltration remains limited by small sample sizes and brief follow-up periods, preventing establishment of definitive conclusions. Future studies with expanded cohorts and extended follow-up are essential to characterize the prognostic features of different molecular subgroups in the context of MELF infiltration.

The prevalence of MELF infiltration in low-grade EC and its association with adverse prognostic factors suggests its value as a biomarker for identifying high-risk patients who may benefit from adjuvant therapy. Currently, postoperative adjuvant therapy for EC is determined primarily by risk stratification without specific modifications based on MELF pattern status. More prospective studies are needed to clarify this question in the future. Nevertheless, identification of MELF pattern should prompt vigilant postoperative surveillance due to its invasive potential. The efficacy of adjuvant treatment in early-stage EC patients with MELF pattern remains controversial. Focused clinical studies are needed to evaluate the risk-benefit profile of adjuvant radiotherapy and to optimize treatment protocols. Well-designed prospective investigations would provide evidence-based recommendations for clinical management and facilitate development of individualized treatment strategies for these patients.

To our knowledge, this meta-analysis provides the most comprehensive evaluation to date of the prognostic significance of MELF invasion pattern in patients with EC. Several limitations must be acknowledged. The retrospective nature of included studies introduces potential confounders that cannot be fully controlled. Heterogeneity among studies likely stems from variations in sample sizes, study designs, patient populations, and adjuvant treatment approaches. The relatively small number of eligible studies limits comprehensive exploration of heterogeneity sources. The molecular characteristics and prognostic implications of MELF invasion in EC remain incompletely characterized due to limited research. Validation of our findings requires additional multicenter, high-quality prospective controlled trials. Current investigations have primarily employed qualitative analysis of MELF invasion pattern. Future studies using quantitative or semi-quantitative methodologies may better elucidate the prognostic significance of MELF invasion in low-grade EC and determine its value in guiding adjuvant therapy decisions.

## 5 Conclusion

This meta-analysis demonstrated a significant association between MELF invasion pattern and increased risks of LVSI and LNM in EC. While MELF pattern was associated with reduced OS and DFS in univariate analyses, this association was not maintained in multivariate analysis. This finding suggests that variables such as surgical approach and adjuvant therapy may modify the impact of MELF pattern on patient outcomes. Considering the limitations of this meta-analysis, additional high-quality, multicenter prospective controlled trials are necessary to confirm these findings and evaluate whether MELF pattern detection should inform adjuvant therapy decisions.

## Data Availability

The original contributions presented in the study are included in the article/**Supplementary Material**. Further inquiries can be directed to the corresponding author.
