# Supplementary material for: Impact of the microcystic, elongated, and fragmented invasion pattern on prognosis in endometrial carcinoma: a comprehensive meta-analysis
Source: Front Oncol. 2025 Jun 25;15:1527324. doi: 10.3389/fonc.2025.1527324 (PMC12237627; doi:10.3389/fonc.2025.1527324)
Supplement: Supplementary file 1 [file DataSheet1.docx]

**Supplementary materials**

**Supplementary material 1** The comprehensive search strategy for PubMed

(("Endometrial Neoplasms"[Mesh]) OR ((((((((((((((((((((Endometrial Neoplasm[Title/Abstract]) OR (Neoplasm, Endometrial[Title/Abstract])) OR (Neoplasms, Endometrial[Title/Abstract])) OR (Endometrial Carcinoma[Title/Abstract])) OR (Carcinoma, Endometrial[Title/Abstract])) OR (Carcinomas, Endometrial[Title/Abstract])) OR (Endometrial Carcinomas[Title/Abstract])) OR (Endometrial Cancer[Title/Abstract])) OR (Cancer, Endometrial[Title/Abstract])) OR (Cancers, Endometrial[Title/Abstract])) OR (Endometrial Cancers[Title/Abstract])) OR (Endometrium Cancer[Title/Abstract])) OR (Cancer, Endometrium[Title/Abstract])) OR (Cancers, Endometrium[Title/Abstract])) OR (Cancer of the Endometrium[Title/Abstract])) OR (Carcinoma of Endometrium[Title/Abstract])) OR (Endometrium Carcinoma[Title/Abstract])) OR (Endometrium Carcinomas[Title/Abstract])) OR (Cancer of Endometrium[Title/Abstract])) OR (Endometrium Cancers[Title/Abstract]))) AND (((microcystic,elongated and fragmented[Title/Abstract]) OR (MELF[Title/Abstract])) OR (Microcystic,elongated,fragmented pattern of invasion[Title/Abstract]))


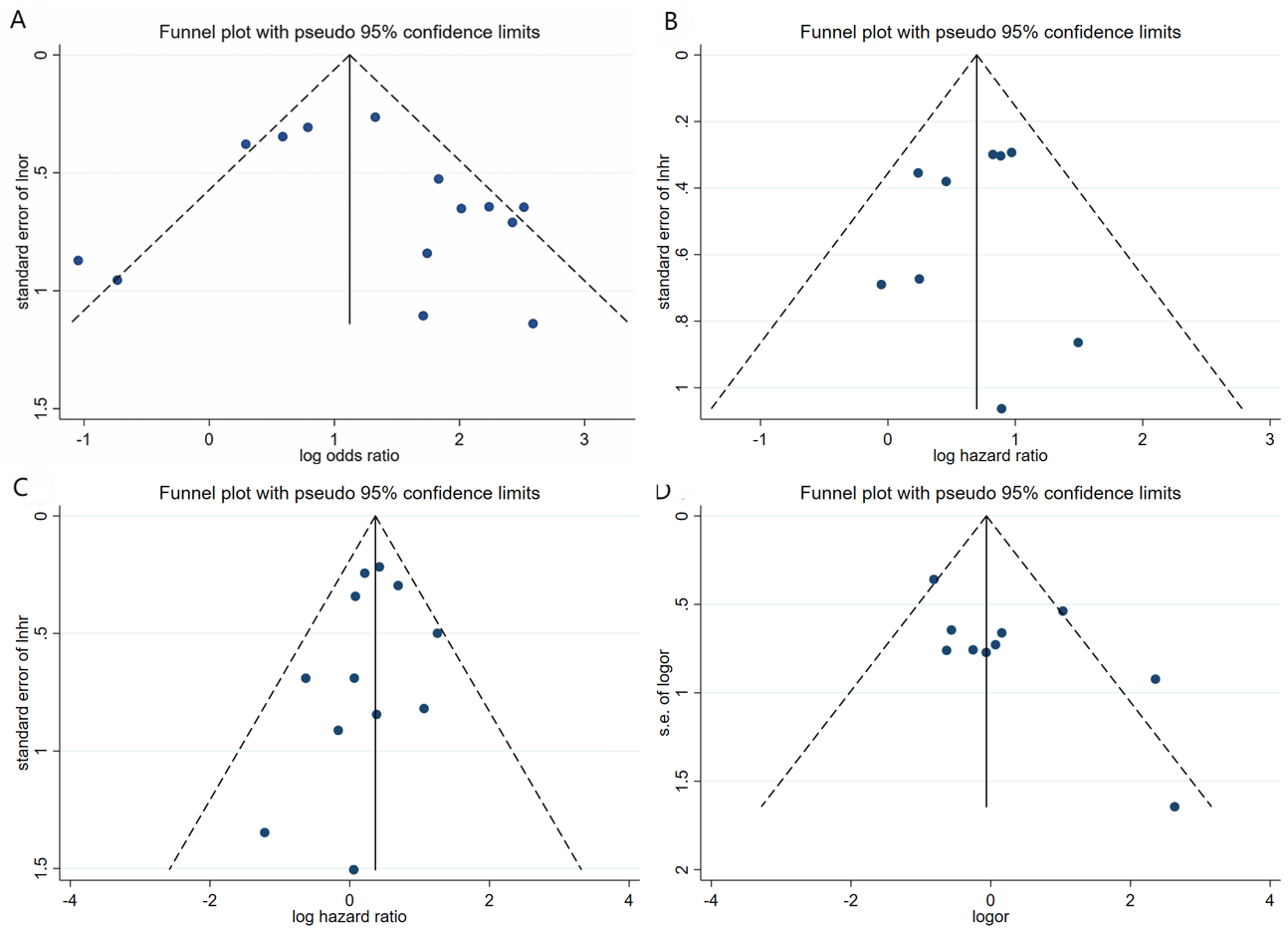


**Supplementary material 2** Funnel plot with pseudo 95% confidence limits of MELF for LNM(A), OS(B) , DFS(C), and RR(D).
